# Modulation of Prrxl1 transcriptional activity by phosphorylation

**DOI:** 10.1186/1753-6561-6-S4-O47

**Published:** 2012-07-09

**Authors:** AS Pessoa, R Soares-dos-Reis, M Falcão, M Matos, CB Monteiro, FA Monteiro, C Reguenga, D Lima

**Affiliations:** 1 Departamento de Biologia Experimental, Faculdade de Medicina, Universidade do Porto, Portugal; 2 IBMC Instituto de Biologia Molecular e Celular, Universidade do Porto, Portugal

## Introduction

Prrxl1 is a homeodomain transcription factor essential for the connectivity and survival of nociceptive neurons in the mouse embryo dorsal root ganglion (DRG) and spinal cord (SC). Prrxl1^-/-^ mice show altered patterning of nociceptive afferent projections to the dorsal SC (dSC), neuronal loss, reduced nociception and failure to thrive. Prrxl1 displays a multiple band pattern on western-blots (WB), which is abrogated by incubation with a phosphatase. Our aim is to further characterize the nature and sites of Prrxl1 post-translational modifications and their impact on its function.

## Methods

Mice were dissected and nuclear extracts of DRG and dSC of mouse embryos (from embryonic day 12.5 to post-natal day 14) were prepared using a Triton-sucrose buffer and analysed by WB. This time-course analysis was further completed by 2D-electrophoresis (isoelectric focusing followed by SDS-PAGE) of embryonic and post-natal dSC extracts. Furthermore, nuclei of ND7/23 cells (a DRG-derived cell-line with nociceptive properties) and embryonic dSC were incubated with a GA^3+^ immobilized metal ion affinity chromatography resin, with affinity for phosphopeptides. Constructs corresponding to truncated versions of Prrxl1 were generated by molecular cloning, and their WB band pattern, transcriptional activity and DNA-binding activity were assessed by luciferase-reporter assays and by a modified DNA pull-down assay. CNBr chemical cleavage of recombinant protein was performed to enhance this analysis. Moreover, site-directed mutagenesis was performed for an evolutionarily conserved putative phosphorylation site which was subjected to the same analysis.

## Results

Prrxl1 is highly phosphorylated and its phosphorylation state varies in a time and tissue specific manner. Phosphorylation seems to occur throughout the protein, but mainly in the N-terminus (encompassing a DNA-binding domain) and in the C-terminus (containing a conserved putative regulatory domain). Evolutionary conserved site analysis revealed a highly conserved phosphorylation site in the homedomain, whose mutation impairs transcriptional activation, but not DNA-binding. Luciferase reporter assays of truncated versions of Prrxl1 have identified the N-terminus as sufficient for the DNA-binding and transcriptional activity of Prrxl1.

## Conclusions

Altogether, our results show that Prrxl1 is mainly phosphorylated in the C-terminus and in the N-terminus. Moreover, these phosphorylations probably play a fundamental role in the regulation of Prrxl1 transcriptional activity in nociceptive neurons.

